# The History of Mind Cure

**Published:** 1888-09

**Authors:** Helen Densmore


### THE HISTORY OF MIND CURE: AN ANALYSIS FROM A PHYSIOLOGICAL STANDPOINT.

BY DR. HELEN DENSMORE.

### PART II.

During the time devoted to this study, I laid aside my preconceived opinions, glad indeed to find a larger truth, if larger truth it proved to be, than the necessity of living a hygienic life in order to secure and maintain health, and for a time I came near accepting their most extreme view: that there is no physiological law, that mind and not physiological law controls the human mechanism; and that to think right thoughts will perform the bodily functions, regardless of the quality of food, air, clothing, etc.

Fortunately for me, I continued my study and observation long enough to find the flaw. I began to see that often patients restored to health by this method fell back, became ill again, and were not so easily cured, if they could be cured at all, the second time. I was taught that to keep from taking cold when exposed to draughts I must deny the power of draughts to give me cold; that I must treat myself.

I was told that the old thought and race belief is very strong, and often we become powerless to stand against it. Then I found the healers themselves succumbing to fits of illness. I called upon one of the most celebrated teachers and healers in Boston, and found that teacher in the throes of cholera morbus. One of my dearest friends and patients who abandoned the hygienic life under which she had been greatly relieved of illness, and which, if she had continued, would have given her permanent health, died of acute peritonitis, after practicing and teaching Mind Cure for over two years with the fervor of a Savanorola.

I used my influence with several of my patients to try the Mental Healing treatment, and in every instance they were urged to give up all thought of hygiene as a remedial agent, and I had only to wait to see those who accepted this advice return to old conditions, and those who did not accept it, but supplemented the hygienic life with the benefits of Mind Cure treatment, were greatly helped by it.

These facts made me pause in making up my mind, as did the facts in regard to the claim of Mental healers when first brought to my attention.

I had learned through the hygienic life that health is man's birthright; that it is as natural to be well as to be born; and I had learned that pathological conditions, all diseases and all tendencies to diseases, are the result of the trangression of hygienic and physiological law; that this statement contains the science of health in a nutshell. Dr. Densmore and I had come to regard health as a moral duty, and we would feel as much ashamed to be ill as to be drunk: and we found ourselves as able to control the making and keeping appointments, in spite of failures from illness, with more certainty than we control any other human event.

All these results and possibilities were to be counted upon only when the laws of health on a hygienic basis were obeyed; and nothing less certain and controllable would satisfy me in exchange for what claimed to be a larger truth and a higher life.

So, when I found illness and return to illness conditioning the new "science," (?) when I found death claiming victims from its ranks, and failures in healing without number to be accounted for; and in seeking the why and wherefore, was told that no promises were ever made as to the *certainty of cure*, or the possibility of attacks of illness: that the *race belief* in sickness, sin and death would sweep in and undo the best thought-work of the individual; and that malicious magnetism is ever on the alert to sieze victims; that it is necessary to live the life enjoined by this new truth all the hours of all the days to be able to be strong enough in it, to make the power available. This, I said, is not a higher truth, when applied to the healing of physical diseases, than the hygienic life, for that will bring valuable results with the certainty of science to every one who will give earnestness and reasonable time to it; and, unlike the Mind Cure practitioner, I always promised this result to my patients if, on their part, obedience to instructions was given; except in cases where actual disorganization of tissue had set in. For myself, I never find it necessary to treat against taking cold, or fear of contagious diseases ; for, in following the physiological life, there is no danger of taking cold, or of contagious diseases. I can, at all times, defy the power of race belief to make me ill ; and so can all those who will learn how to live and live it. So, I say, I called a halt in the march away from my intrenchments, and began a new survey of the battlefield whereon the armies of sin, sickness and death are engaged in deadly warfare against the human race.

An earnest study of the metaphysical method of curing diseases convinces me that it contains a great and valuable truth ; that no one can earnestly study it without finding it, and, on applying it to their lives, will be greatly helped and enriched by it. But it has not destroyed my belief in the value of an orderly physiological life, and that when the teachers of Christian science tell us there is no body, no pain, no disease, and then offer their services to cure—no matter what they call it, belief, illusion, delusion, false reports of matter, etc., " a rose by any other name," —to be paid for as other physicians are paid ; that, when they eat, drink, sleep and clothe themselves, according to the need of the climate and state of the body, they are guilty of gross inconsistency, and prove their art is not a science ; indeed, it has no resemblance to science.

It is plain to me that if Mental Healing ever becomes what it claims to be, in any true sense, a science, it will have to change front completely on this subject ; as Mrs. Grimké says, "The Mental healers who say it makes no difference what we eat and drink, are blind leaders of the blind, and are sure to be all ditched together, unless their eyes are opened." Mrs. Grimké adds, " the reason why I consider it does make a difference what we eat, has a spiritual and not physical basis." That all manifestations in time and space have a spiritual basis must be believed by all who are not rank materialists, no matter whether the whys and wherefores are comprehended or not.

The transcendent value of Christian science, with all its crudities, rests in the fact that it has the tendency to startle the minds of men from the growing materialistic tendencies of the age. But with its crude formulation it stands open to intelligent criticism as well as to the unnecessary and often unreasoning ridicule of the schools of material science. That it has swept down upon the fierce skepticism of the age and found the logdment that it has, nothwithstanding the nonsense prattled about it, proves it has a foundation in a powerful half truth.

At a lunch party where Mrs. Stuart was present one of the guests refused cheese, for physiological reasons ; whereupon, it is reported, Mrs.

Stuart, with a contemptuous expression, exclaimed, "Think of an immortal soul being dominated by a piece of cheese!" No one present seems to have been astute enough to ask this lady: "When we cannot read in the dark without clairvoyant vision, or be buried in live coals, or go out into the winter blasts unclad, without suffering the usual results of the transgression of the laws of time and space, does it prove that the immortal soul is dominated by these conditions?"

It is quite true that man's spirit, in exceptional instances, is able to transcend physiological law, and to enable him to swallow poisons without any apparent physical effect. But thoughtful students of Mental Healing will perceive that this is not the ordinary law of life in the body; and while it is true that a quart of whiskey, in exceptional instances, may be taken with no apparent physical harm, it is also true that neither Mrs. Stuart nor any other mental healer is able to partake of excessive quantities of alcoholic drink daily, without manifesting the same results that follow its general use. Just so, unphysiological foods may be taken and no immediate deleterious results appear, but just as surely as "the soul" of a Mental healer will be dominated by an excessive indulgence in alcoholic drinks, so will it be if unphysiological foods are habitually used. A drink of whiskey is more unphysiological than a piece of cheese, and "the soul" is more apt to be dominated by whiskey than cheese, but only in the ratio of its unwholesomeness.

When Mrs. Stuart declares that there is no physiological law she must be prepared to show that we can live without food. It is a law of physiology that animal life is sustained by food, and this food must be directly or indirectly obtained from the vegetable kingdom; and that the heat of the body is created by carbon. If thought carries on the processes of digestion and assimilation, outside of physiological action, then powdered stone coal can be used by the human economy to produce that heat. Will any Mind Curer claim that powdered coal is capable of being used to make the necessary heat of the body?

But the absurdity of the whole matter rests in the mistake that is made in supposing that the soul can be dominated at all. These teachers declare the real man (soul or spirit) is always right; that all is spirit; there is no matter: all manifestations of the body are illusions, shadows, nothingnesses; and consequently they arrive at the great central truth of the system, there is *no evil*: for all forms of evils are the shadows, the false reports of the senses; and to deny sin, sickness and death as realities, and place the thought of truth, the nothingness of matter, in the place of this falsity, is the first step in the treatment of disease.

According to this philosophy the soul is not disturbed, but rests in the absolute, without fear or possibility of being dominated by any condition of its expression in visibility.

But it matters not for the practical work before us, whether matter be, as considered by the idealists, nothing, having existence alone in our consciousness, or whether it be a something which spirit uses, moulds, forms. As Emerson says, "To pure spirit matter is fluid, it is volatile, it is obedient."

So I say whichever it be, matter, as having existence only in consciousness, or whether it be a something which exists as the expression of spirit, its servant and not master, for all practical purposes, is unimportant. That it exists for all practical uses is undeniable. The visible universe, physics and chemistry, we treat from the sense world as external in their manifestations; and the law that governs in this realm is not the cause of things, but the effect of a cause back of all phenomena. The poisons that compromise physical life act in accordance with physiological law, which law is also an effect of a cause which lies back of all expression; and such phenomena are not claimed by the true spiritual philosopher as causation. The law of gravitation is not a cause, but an effect, and set in motion by the universal supreme cause. The law of gravitation does not move the heavenly bodies; but the heavenly bodies move in accordance with that law, which is itself the result of Supreme Cause.

So transgression of physiological law is not the cause of disease, but disease comes through such transgression, in accordance with the mandate of that Supreme Cause of all visibility; i. e., manifestations of spirit in time and space, resulting in the forms of nature, the idea of God, or the creative will of the universal spirit.

So when Mental healers tell their patients that there is no physiological law, that there are no bodily functions; no blood; no digestive apparatus; no lungs; no heart; that they are to eat anything at any time they fancy, *not to fear* being the only care to be taken, they do a grievous wrong. If a man steps from the roof of a high building he falls to the earth and is injured in accordance to the laws of gravitation in the ratio of the distance which he falls, and the nature of the substance which he falls upon. So, too, when a man disobeys physiological law, he is damaged; for instance, if he habitually partakes of food not naturally adapted to the human organism, he is damaged in accordance with the law of physiology in the ratio of the transgression.

The earnest student of Mental Science will find that cheese is not a natural, wholesome or desirable food, as he will find that whiskey is not a natural, wholesome, or desirable drink ; and that those persons who will persevere in eating cheese or drinking whiskey will be injured in the ratio of the transgression. In neither case will the soul be dominated, either by the cheese or the whiskey ; nevertheless, it is as absurd to counsel the disobedience of physiological law, as to rebel against the law of gravitation ; and it is as undesirable, in the language of Mental Healing science, to "overcome the fear of cheese" as to "overcome the fear of whiskey." There should be no fear in either case ; knowledge is all that is needed. As the Mental healers teach, a knowledge of the truth of Being raises us above the reign of fear, for when we *know* God, and obey His laws, we are at-one-ment with him of necessity, including the law of gravitation, physiology, and all the laws governing the manifestations of spirit in time and space : and these are His laws, all the time remember, and not man's laws.

I am all the more earnest in this matter, considering the very great importance that Mental Healing is to the world, and the boon that it is destined to prove, providing it is not permanently handicapped by the ridiculously absurd statements of many of its devotees.

We live in an age of gross materialism, and that which is called science is doing much to drown the voice of the spirit. The great truth of the supremacy of spirit is struggling with this power ; and the phenomenon of Mind Cure, Mental Healing, Christian Science, or by whatever name the movement is known, is a most valuable and important contribution ; and I am solicitous that its usefulness shall not be neutralized by the follies and ignorance of its formulators and practitioners.

The practical work that it has done, and is doing, is very great. Before its advent there were multitudes of persons shamelessly cultivating their physical ills, with a mistaken notion that it made them interesting, and from an overweening craving for personal sympathy. The Mind Cure movement has registered its beneficial influence in a perceptible decrease of this tendency on the part of invalids. The shamefulness of illness is now understood by thousands who were before entirely ignorant of it. Its beneficial effects are also seen in a perceptible emancipation of thousands from the domination of the doctor. Ignorance of the power of the spirit to cure disease and of the healing power of nature, as taught by the ancients, is one of the most, if not the most lamentable, of our age. Mind Cure has taught that to call the doctor when a person is taken ill, is unnecessary ; that all that is required is to have no fear, recognize the spirit within, and healing will surely follow; and the super stition that is at the foundation of the employment of a doctor has received a wholesome check.

The benefit is not confined to the avoidance of drugs alone ; there is a most dense ignorance abroad in the world concerning food, on the part of the physician and layman alike. When one is attacked with an acute illness, nature generally destroys appetite for food ; but the doctor and attendant, possessed of the absurd fear of resultant weakness, insists that the patient shall eat whether there be appetite or no. There will come a time when all will know that the most important thing to be done in acute illness is abstinence from food. One of the effects of the Mental Healing movement is to aid in emancipating many from the fear of fasting, and to rely upon the healing power of nature—God.

Over and above all this, the Mind Cure movement has been to thousands glad tidings of great joy—a voice from the realm of the real. It has made Christianity a verity to many who were before living in the letter of it alone. All along the ages God's inspired oracles from the mountain tops of inspiration have heralded to man the eternal verities of the spirit and the nothingness of the material. This movement joins forces with the armies of the Lord in the past, and is another and much needed manifestation from the realm of the eternal.

It is because of the importance of this movement and its possibilities for good that I deprecate its follies. I earnestly pray that a time may come in its history, and that it may not be long deferred, when, instead of teaching disobedience to nature's laws, instead of ignoring an orderly hygienic life, and in so doing divorcing what seems to me ought to be divinely joined, they will awake before it is too late, and teach in connection with the supremacy of spirit, obedience to the laws set in motion by that Universal Spirit, which is their source and cause.

